# Lung anatomy, energy load, and ventilator-induced lung injury

**DOI:** 10.1186/s40635-015-0070-1

**Published:** 2015-12-15

**Authors:** Alessandro Protti, Davide T. Andreis, Marta Milesi, Giacomo E. Iapichino, Massimo Monti, Beatrice Comini, Paola Pugni, Valentina Melis, Alessandro Santini, Daniele Dondossola, Stefano Gatti, Luciano Lombardi, Emiliano Votta, Eleonora Carlesso, Luciano Gattinoni

**Affiliations:** Dipartimento di Anestesia, Rianimazione ed Emergenza Urgenza, Fondazione IRCCS Ca’ Granda-Ospedale Maggiore Policlinico di Milano, Milan, Italy; Dipartimento di Fisiopatologia Medico-Chirurgica e dei Trapianti, Università degli Studi di Milano, Via Francesco Sforza 35, 20122 Milan, Italy; Centro di Ricerche Chirurgiche Precliniche, Fondazione IRCCS Ca’ Granda-Ospedale Maggiore Policlinico, Università degli Studi di Milano, Milan, Italy; Dipartimento dei Servizi, Radiologia, Fondazione IRCCS Ca’ Granda—Ospedale Maggiore Policlinico, Milan, Italy; Dipartimento di Elettronica, Informazione e Bioingegneria, Politecnico di Milano, Milan, Italy

**Keywords:** Mechanical ventilation, Ventilator-induced lung injury, Lung stress and strain, Inspiratory capacity, Energy load, Experimental animal model

## Abstract

**Background:**

High tidal volume can cause ventilator-induced lung injury (VILI), but positive end-expiratory pressure (PEEP) is thought to be protective. We aimed to find the volumetric VILI threshold and see whether PEEP is protective *per se* or indirectly.

**Methods:**

In 76 pigs (22 ± 2 kg), we examined the lower and upper limits (30.9–59.7 mL/kg) of inspiratory capacity by computed tomography (CT) scan at 45 cmH_2_O pressure. The pigs underwent a 54-h mechanical ventilation with a global strain ((tidal volume (dynamic) + PEEP volume (static))/functional residual capacity) from 0.45 to 5.56. The dynamic strain ranged from 18 to 100 % of global strain. Twenty-nine pigs were ventilated with end-inspiratory volumes below the lower limit of inspiratory capacity (group “Below”), 38 within (group “Within”), and 9 above (group “Above”). VILI was defined as death and/or increased lung weight.

**Results:**

“Below” pigs did not develop VILI; “Within” pigs developed lung edema, and 52 % died before the end of the experiment. The amount of edema was significantly related to dynamic strain (edema 188–153 × dynamic strain, *R*^2^ = 0.48, *p* < 0.0001). In the “Above” group, 66 % of the pigs rapidly died but lung weight did not increase significantly. In pigs ventilated with similar tidal volume adding PEEP significantly increased mortality.

**Conclusions:**

The threshold for VILI is the lower limit of inspiratory capacity. Below this threshold, VILI does not occur. Within these limits, severe/lethal VILI occurs depending on the dynamic component. Above inspiratory capacity stress at rupture may occur. In healthy lungs, PEEP is protective only if associated with a reduced tidal volume; otherwise, it has no effect or is harmful.

## Background

Various forms of ventilator-induced lung injury (VILI) have been described since the definition of adult respiratory distress syndrome (ARDS) [1]. Barotrauma was the first to be recognized as a form of stress at rupture, leading to pneumothorax, pneumoperitoneum [2], etc. In subsequent years the concept of volutrauma (excessive strain) [3] emerged, and years later, atelectrauma and its inflammatory reaction was recognized, first of all ex-vivo [4, 5] then later in clinical settings [6]. Excessive tidal volume is now recognized as the first cause of VILI [7], supporting the concept of volutrauma, while limiting airway pressure to ≈30 cmH_2_O in patients with normal chest wall is used as a surrogate of the maximal tolerable stress (transpulmonary pressure).

The protective effects of positive end-expiratory pressure (PEEP) on VILI were described by Webb and Tierney [8] in their seminal experiments. Although dramatically effective in experimental ARDS, in human ARDS, higher PEEP failed to show clear benefits over lower PEEP [9–11]. Possible benefits were only suggested by meta-analysis in the subgroups of the most severe ARDS patients [12, 13]. Therefore, while the relation between higher tidal volume and VILI is robust and largely accepted, the protective effects of PEEP are not so widely agreed.

The tidal change in lung volume is associated with a cyclic energy load to the respiratory system; the energy being equal to the pressure applied multiplied by the change in volume (*P* × dV), summed along the inspiratory volume-pressure curve. In contrast, PEEP, once applied, does not impose any cyclic energy load as the volume is constant (dV = 0). Therefore, considering both the lung volume distortion and the energy load, we set out to answer the following questions: Is there a threshold of volume distortion/energy load producing VILI? Is the application of PEEP protective *per se* or is it just an indirect effect, due to the concomitant reduction in tidal volume when it is applied?

Over the years, we have conducted a series of long-term animal experiments in which we tested different tidal volumes (dynamic strain), transpulmonary pressure (dynamic stress), and levels of PEEP (static strain and static stress) [14, 15]. Considering these different experiments together, we concluded that the energy/power load provided a single explanation of the different phenomena. Therefore, we reanalyzed data not previously published from previous experiments and added further experiments to cover a wider range of tidal volumes and PEEP levels. This enabled us to define the interaction between the anatomical limits of lung expansion, as assessed by computed tomography (CT) scan, the inspiratory volumes applied (tidal volume + PEEP volume), and the dynamic and static energy used to induce VILI in healthy animals. Therefore, our aim here is to advance a unifying theory for VILI in the healthy lung, from old and new studies taken together.

## Methods

### Ethics, consent, and permissions

This study was approved by the local ethical review board (Ministero della Salute, Direzione Generale della Sanità Animale e dei farmaci veterinari, Rome, Italy); it was conducted according to the Declaration of Helsinki for the use and care of animals and complied with international recommendations [National Research Council (U.S.), Institute for Laboratory Animal Research (U.S.), National Academies Press (U.S.): Guide for the care and use of laboratory animals, 8th ed., Washington, D.C, National Academies Press, 2011].

### Experimental procedure

The study population consisted of 76 anesthetized (propofol and medetomidine iv) and paralyzed (pancuronium bromide iv) healthy pigs (22 ± 2 kg). Forty-five had already been included in 2 previous studies [14, 15] and 31 were specifically added for the present work. At baseline, the pigs underwent a whole-lung CT scan at 0 and 45 cmH_2_O of constant airway pressure. An additional whole-lung CT scan was done at the clinical PEEP in pigs ventilated with end-expiratory pressure higher than 0 cmH_2_O. The respiratory system and lung volume-pressure curves were obtained starting from functional residual capacity (FRC) (airway pressure 0 cmH_2_O), in 100-mL steps in 43 of the 76 animals.

We defined global strain as the ratio between the total end-inspiratory volume (tidal volume + PEEP volume) and the FRC, measured with the lung CT scan. We defined dynamic strain as the strain due to tidal volume (tidal volume/FRC) and static strain as the strain due to PEEP (PEEP volume/FRC).

Twenty-nine pigs [14] were ventilated at PEEP 0 cmH_2_O with a tidal volume producing a dynamic strain between 0.45 and 3.3, to identify the “lethal strain,” which was found to be greater than 2.5. In these pigs, as no PEEP was applied, the global strain was equal to the dynamic strain. Sixteen pigs [15] were treated with a global strain of 2.5 resulting from different proportions of tidal volume (dynamic strain) and volume due to PEEP (static strain) to investigate the protective effect of PEEP. Thirty-one pigs (the ones added in this study, calculating the sample size on the basis of our previous experience) were ventilated with different tidal volumes and PEEP combinations to cover the dynamic/static strain combinations not explored in the two earlier studies.

**Fig. 1:**
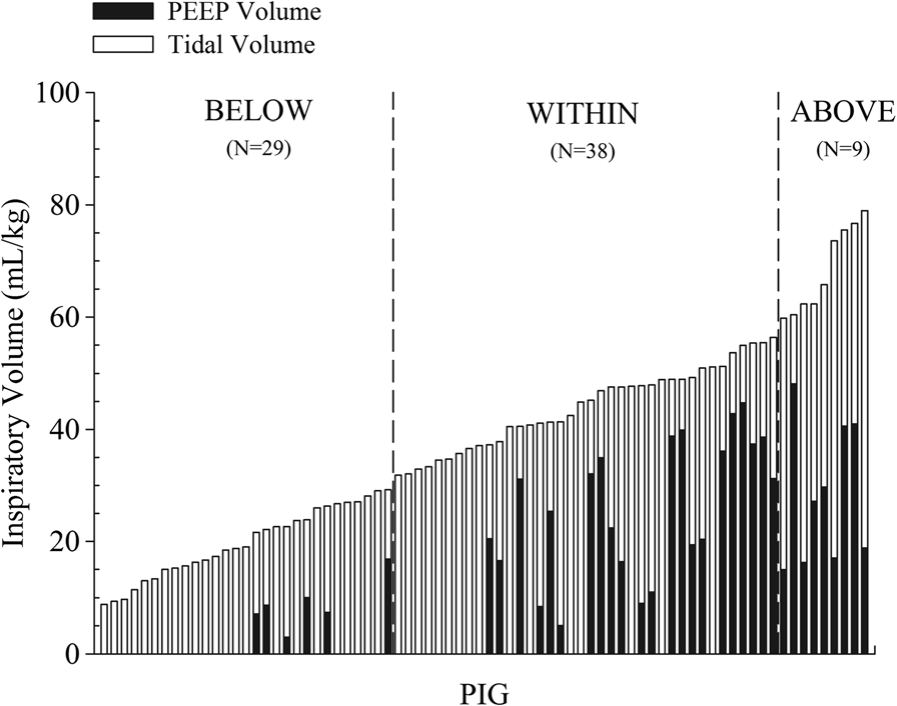
Global end-inspiratory volume and the different dynamic and static proportions. The distribution of global end-inspiratory volumes used in this study is shown with the different proportions of dynamic (tidal volume, *white bars*) and static components (*black bars*). Pigs were grouped according to end-inspiratory volume lower (BELOW), within (WITHIN), or higher (ABOVE) than normal inspiratory capacity (*vertical dashed lines*)

The global strain applied in the 76 pigs ranged from 0.45 to 5.56. Dynamic strain ranged from 100 to 18 % of global strain. Figure 1 summarizes the global end-inspiratory volume (tidal volume + PEEP volume) and the different dynamic and static proportions. The inspiratory/expiratory time ratio was kept between 1:2 and 1:3, the respiratory rate was set at 15 bpm, and the fraction of inspired oxygen was 0.5. All animals were instrumented with an endotracheal tube, arterial, central venous, pulmonary artery, urinary, and esophageal catheters and kept in anesthesia and paralysis. The experiments were terminated after 54 h or sooner if animals died.

### Measurements

**Fig. 2:**
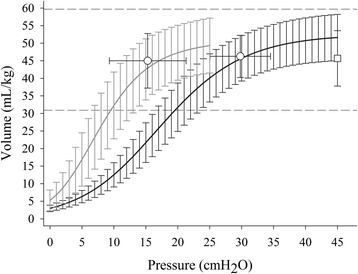
Respiratory system and lung pressure-volume curve. Mean (±standard deviation) pressure-volume curve of the total respiratory system (*black line*) and the lung (*gray line*) obtained by sigmoidal fitting (see text for method). The *white dots* indicate the upper corner points and the *white squares* the average inspiratory capacity (TLC-FRC) obtained by CT scan at 45 cmH_2_O airway pressure. *Horizontal dashed lines* indicate the 95 % confidence limits of inspiratory capacity (mean ±2 standard deviations)

Lung weight, FRC (gas content at 0 cmH_2_O PEEP), total lung capacity (TLC, gas content at 45 cmH_2_O), and PEEP volume ((end-expiratory gas content with PEEP)-FRC) were measured with quantitative CT scan analysis. The fractions of over, normally, poorly, and not inflated lung tissue were computed using standard CT thresholds (−900, −500, −100 Hounsfield units (HU) [16]). Overall mean pressure-volume curves of the whole respiratory system and of the lung were obtained by averaging the volumes calculated from the individual fittings of the 100-mL steps curves obtained in 43 animals. Volumes were taken at 1 cmH_2_O pressure intervals from 0 to 45 cmH_2_O of airway pressure and from 0 to 25 cmH_2_O of transpulmonary pressure (Fig. 2). The individual curves were fitted with a sigmoidal equation:

$$ V=a + \left[\frac{b}{1+{e}^{-\left(P-c\right)/d}}\right] $$

The upper inflection point (*UIP*,_,_ the pressures at which the slope rapidly changes) can be defined as UIP = *c* + 2*d* and the maximal compliance (at the “most linear” portion of the pressure-volume (PV) curve) is b/4d [17].

#### Inspiratory capacity

The inspiratory capacity was measured in each piglet as the difference between TLC and FRC. TLC and FRC were measured with CT scan as the gas content at 45 and 0 cmH_2_O, respectively. We used the average inspiratory capacity ±2 standard deviations as a reference value to which the individual pigs were compared. Airway and transpulmonary pressures corresponding to the upper and lower limits of normal inspiratory capacity were taken from the volume-pressure curves.

#### Energy load

**Fig. 3:**
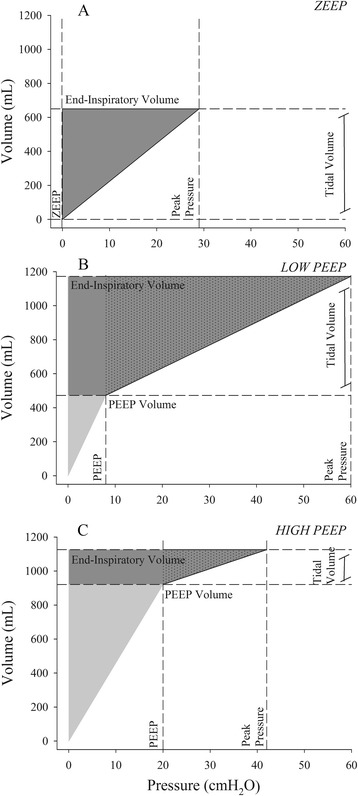
Energy calculation. Three examples of energy calculation in the following: **a** a pig ventilated with PEEP 0 cmH_2_O, **b** a pig ventilated with low PEEP (8 cmH_2_O), and **c** a pig ventilated at high PEEP (20 cmH_2_O). The energy load is composed of a static (when PEEP is higher than 0 cmH_2_O) and a dynamic contribution: global energy load = static energy load + dynamic energy load. Static energy load = [PEEP × PEEP volume/2] (*light gray triangles*). Dynamic energy load = (Peak pressure − PEEP) × TV/2 + (PEEP × TV) = [(PEEP + Peak pressure) × tidal volume / 2] (*dark gray triangles* (panel **a**) or trapezoids (panels **b** and **c**)). The *dark gray trapezoids* (panel **b**, **c**) are composed of a triangle (*black dotted area*), and a rectangle. The triangle represents the term (Peak pressure − PEEP) × TV/2, due to cyclic tidal breath; the rectangle represents the term (PEEP × TV) due to the ventilation (volume change) starting from a pressure level higher than zero (PEEP). *Vertical dashed lines* indicate PEEP and peak pressures; *horizontal dashed lines* PEEP volume and end-inspiratory volume (tidal volume is the difference between the two)

The energy load to the respiratory system (Fig. 3) comprises a static component, due to PEEP and PEEP volume (conceptually equivalent to potential energy), and a dynamic cyclic component, due to driving pressure and tidal volume above PEEP (conceptually equivalent to kinetic energy). As a rough simplification:

$$ \mathrm{Static}\ \mathrm{energy}\ \mathrm{load} = \left(\mathrm{PEEP}\times \mathrm{PEEP}\ \mathrm{volume}\right)\ /2 $$

$$ \begin{array}{l}\mathrm{Dynamic}\ \mathrm{energy}\ \mathrm{load} = \left(\mathrm{Peak}\ \mathrm{pressure} - \mathrm{PEEP}\right)\times \mathrm{T}\mathrm{V}/2 + \left(\mathrm{PEEP}\times \mathrm{T}\mathrm{V}\right)\ \\ {}\kern9.01em  = \left(\mathrm{PEEP} + \mathrm{Peak}\ \mathrm{pressure}\right)\times \mathrm{T}\mathrm{V}/2\end{array} $$

This equation computes the area of the dark gray trapezoid (Fig. 3), where the value of Peak pressure represents the major base, the value of PEEP is the minor base, and the tidal volume represents the height. The area of the trapezoid is the sum of the areas of a “triangle,” due to the cyclic tidal breath and to the pressure variation Peak pressure − PEEP only, independent on the “starting value” (PEEP), and of a “rectangle” associated to the lung volume variation starting from a pressure level higher than zero (PEEP).

The energy load computed this way underestimates the energy actually applied cyclically as it does not account for the pressure spent for gas movement, the surface tension forces and tissue resistances to motion. The static energy, after the first application of PEEP, does not imply any further energy load to the respiratory system. During ventilation, the cyclic load to the lung parenchyma is only due to the dynamic energy, although the total energy in the system includes both its static and dynamic components.

### Data analysis and outcome measures

The effects of tidal volume/dynamic strain, airway pressure/stress, and the effects of PEEP on VILI of the single animals were studied using, as a reference, the anatomical limits of the whole population (the upper and lower limits of normal inspiratory capacity). The individual animals were therefore classified as below, within and above groups referring to the whole population studied:

“Below” group—29 pigs globally inflated with a volume of gas smaller than the lower limit of normal inspiratory capacity (ventilation below inspiratory capacity).

“Within” group—38 pigs globally inflated with a volume of gas between the lower and upper limits of normal inspiratory capacity (ventilation at inspiratory capacity).

“Above” group—9 pigs globally inflated with a volume of gas larger than the upper limit of normal inspiratory capacity (ventilation above inspiratory capacity).

VILI was defined as either death or edema. This injury may manifest rapidly as stress at rupture with massive pneumothorax or, more slowly, as progressive edema. Therefore, we considered the “lethal ventilation” and lung edema as outcome measures. The “lethal ventilation” included pigs that died before the scheduled 54 h of the experiment because of gross pneumothorax or lung edema so severe as to prevent survival. Lung edema compatible with survival at 54 h is a less severe form of VILI. Lung edema was estimated as the difference between the lung weight directly measured at autopsy at the end of the experiment and lung weight measured by CT scan at baseline.

To clarify better whether PEEP was directly protective on VILI or its protective effect was due to the reduction in tidal volume, we examined the three groups of pigs, as above: animals in which the tidal volume (not the total end-inspiratory volume) was below, within, or higher than the boundaries of normal inspiratory capacity. As only one pig had a tidal volume exceeding the upper limit, we analyzed only two groups, one in which the tidal volume (dynamic strain) was below the lower limit of inspiratory capacity (47 pigs) and one (29 pigs) in which the tidal volume was above the lower limit.

### Statistical analysis

Results are reported as mean ± standard deviation. Continuous data were analyzed with Student’s *t* test, Wilcoxon’s signed rank test, as appropriate, and two-way analysis of variance (ANOVA) (on ranks when appropriate). Associations between variables were analyzed with linear regression. Categorical data were compared with the chi-square test. A two-tailed *p* < 0.05 indicated statistical significance (Sigma Plot 11, Jandel Scientific Software; San Jose, CA).

## Results

The baseline characteristics of the respiratory system of these 76 healthy pigs, measured in prone position during anesthesia and muscle relaxation, are summarized in Table 1. The lower and upper limits of normal inspiratory capacity were 30.9 and 59.7 mL/kg body weight, i.e., two standard deviations from the mean. The upper and lower limits of airway pressure were 20.3 and 39.3 cmH_2_O, i.e., also two standard deviations from the mean.

**Table 1:** Baseline lung characteristics

Lung weight (g)	321 ± 40
Functional residual capacity (FRC) (mL)	388 ± 93
Inspiratory capacity (mL)	968 ± 165
Inspiratory capacity (mL/kg)	45.3 ± 7.2
Total lung capacity (mL)	1357 ± 225
Total lung capacity (mL/kg)	63.4 ± 9.9
Upper inflection point volume (mL)	991 ± 147
Upper inflection point airway pressure (cmH_2_O)	29.8 ± 4.7
Maximal total respiratory system elastance (cmH_2_O/L)	22.4 ± 5.0
Specific lung elastance (cmH_2_O)	6.72 ± 3.07
Non-aerated tissue at FRC (g (%))	17 ± 13 (5 % ± 3)
Poorly aerated tissue at FRC (g (%))	117 ± 61 (36 % ± 17)
Well-aerated tissue at FRC (g (%))	186 ± 52 (59 % ± 17)
Over-aerated tissue at FRC (g (%))	0.02 ± 0.04 (0 % ± 0)

Baseline lung characteristics obtained by computed tomography (before the start of the experiment, 73 pigs) and PV curve (43 pigs). Functional residual capacity was the volume of gas at 0 cmH_2_O of airway pressure. Total lung capacity was the volume of gas at 45 cmH_2_O. Maximal total respiratory system elastance was obtained at the maximal slope of the PV curve. Specific lung elastance was computed as the ratio of global stress to global strain. Values are mean ± standard deviation

**Fig. 4:**
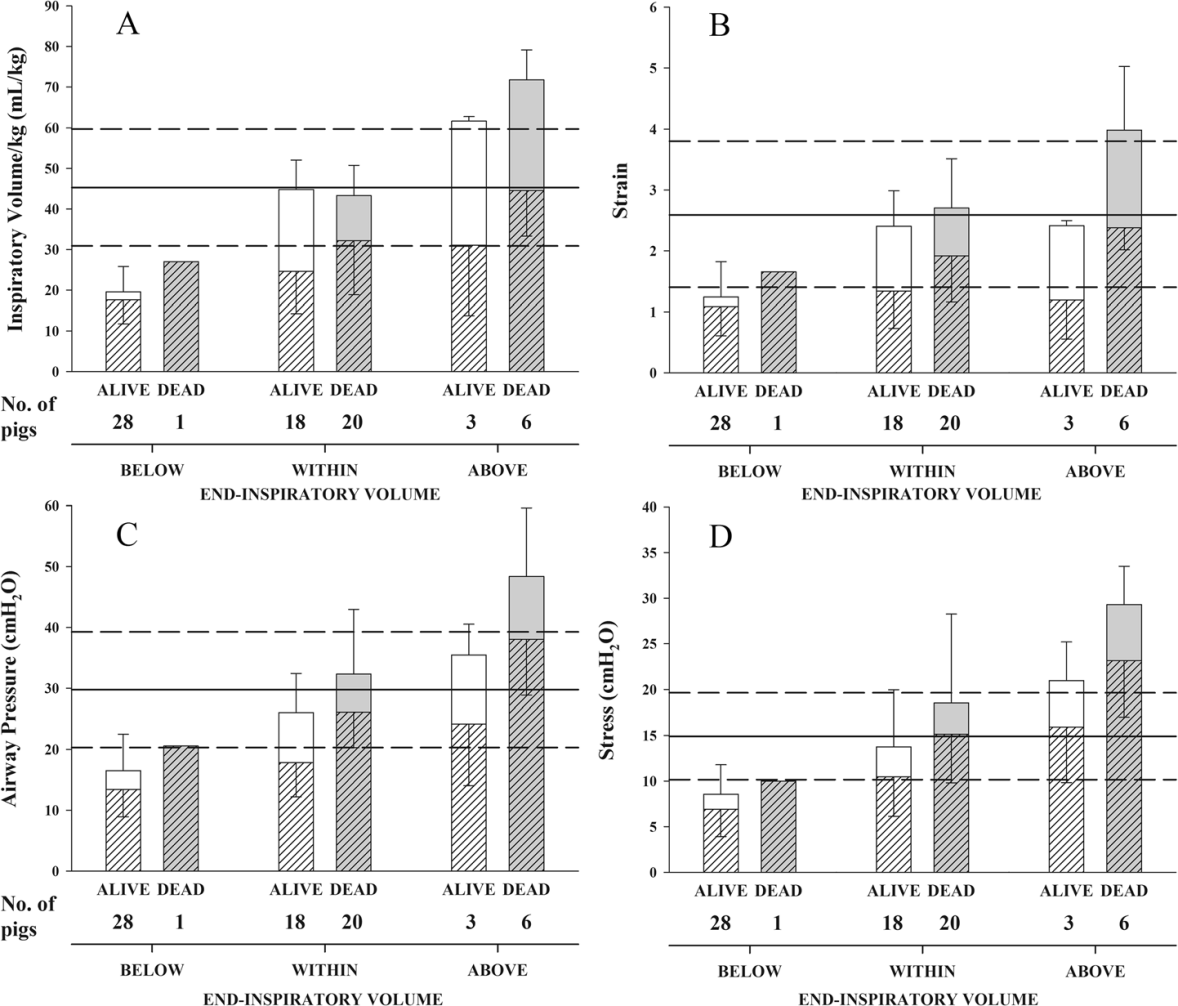
Outcomes in pigs according to end-inspiratory volumes. Mean(± standard deviation) for different variables. Pigs were grouped according to normalized inspiratory volume lower (BELOW), within (WITHIN), or higher (ABOVE) than normal inspiratory capacity and according to outcome: ALIVE (*white bars*) or DEAD (*gray bars*). The *whole bar* indicates a dynamic (coarse stack) and a static component (no pattern). A *solid horizontal line* represents the average reference value; *medium-dashed lines* represent mean ± 2 standard deviations reference values, i.e., the lower and upper limits of the variable. Statistical analysis: two-way ANOVA or on ranks, as appropriate (fixed effects: inspiratory volume and outcome). **a** Total end-inspiratory volume (mL/kg, *whole bar*) (Inspiratory volume *P* < 0.001, Outcome *P* = 0.073, Interaction = 0.067), tidal volume (mL/kg, coarse pattern) (Inspiratory volume *P* = 0.106, Outcome *P* = 0.040, Interaction *P* = 0.908), PEEP volume (mL/kg, no pattern) (Inspiratory volume *P* < 0.001, Outcome *P* = 0.306, Interaction *P* = 0.537). Reference value: inspiratory capacity (CT scan) normalized on body weight. **b** Total strain (*whole bar*) (Inspiratory volume *P* < 0.001, Outcome *P* = 0.059, Interaction *P* = 0.245), dynamic strain (coarse pattern) (Inspiratory volume *P* = 0.536, Outcome *P* = 0.004, Interaction *P* = 0.428), static strain (no pattern) (Inspiratory volume *P* = 0.006, Outcome *P* = 0.500, Interaction *P* = 0.438). Reference value: inspiratory capacity (CT scan) on FRC. **c** Absolute plateau airway pressure (*whole bar*) (Inspiratory volume *P* < 0.001, Outcome *P* = 0.107, Interaction *P* = 0.970), plateau airway pressure minus PEEP (coarse pattern) (Inspiratory volume *P* < 0.001, Outcome *P* < 0.001, Interaction *P* = 0.437), PEEP (no pattern) (Inspiratory volume *P* = 0.024, Outcome *P* = 0.271, Interaction *P* = 0.723). Reference value: airway pressure at upper inflation point (PV curve). **d** Total stress (*whole bar*) (Inspiratory volume *P* < 0.001, Outcome *P* = 0.797, Interaction *P* = 0.344), dynamic stress (coarse pattern) (Inspiratory volume *P* < 0.001, Outcome *P* = 0.092, Interaction *P* = 0.330), static stress (no pattern) (Inspiratory volume *P* = 0.014, Outcome *P* = 0.474, Interaction *P* = 0.756). Reference value: airway pressure reference values multiplied by the ratio of transpulmonary pressure at the upper inflation point (PV curve) to airway pressure at the upper inflation point

Figure 4 shows the outcome of the pigs ventilated with end-inspiratory volumes (tidal volume plus PEEP volume) below, within, and above the limits of normal inspiratory capacity. The upper panels present the inspiratory volume (A) and associated strain (B), and the lower panels show the airway (C) and transpulmonary pressures (stress, D). Mechanical ventilation was lethal (at the rate of 15 bpm) only when end-inspiratory volumes were within or above the limits of normal inspiratory capacity (“Within” and “Above” groups). The contribution of the tidal volume and PEEP volume to the total end-inspiratory volume seems to play a significant role too. Panels a and b of Fig. 4 show that out of the 38 animals which had similar total end-inspiratory volumes, within the limits of inspiratory capacity, the 20 that finally died had significantly larger tidal volumes (dynamic strain) than the 18 that survived (32.2 ± 13.3 mL/kg and 24.7 ± 10.4 mL/kg, *P* = 0.03). The PEEP volume tended to be lower, though not significantly so, in non-survivors than survivors (11.1 ± 16.6 mL/kg and 20.2 ± 14.7 mL/kg, *P* = 0.06).

**Fig. 5:**
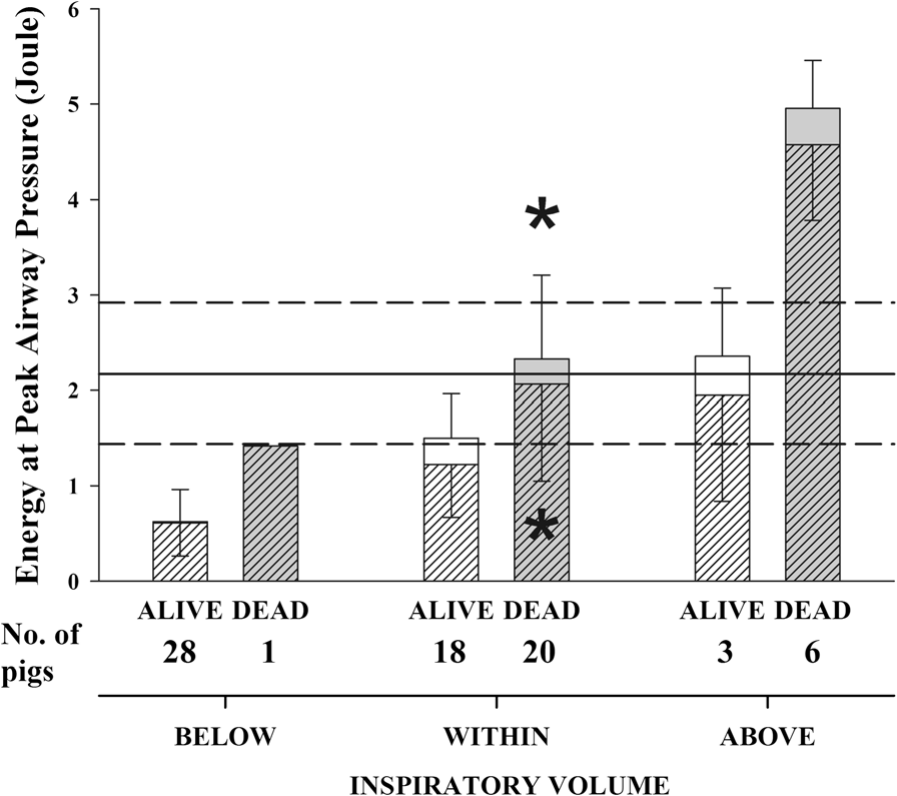
Outcomes of pigs and energy at peak airway pressure. Mean (± standard deviation) energy at peak airway pressure, expressed as joule. Pigs were grouped according to normalized inspiratory volume lower (BELOW), within (WITHIN) or higher (ABOVE) than normal inspiratory capacity. Pigs were also divided according to outcome: ALIVE (white bars) or DEAD (gray bars). The whole bar is composed of a dynamic component (coarse stack) and a static component (the stack with no pattern). A solid horizontal line indicates the average reference value of energy at 45 cmH_2_O airway pressure, medium-dashed lines represent mean ± 2 standard deviation reference values, i.e. the lower and the upper limits of the variable. Statistical analysis: two-way ANOVA on ranks (fixed effects: Inspiratory volume and Outcome). Total energy at peak airway pressure (entire bar) *(Inspiratory volume P < 0.001, Outcome P < 0.001, Interaction = 0.804)*, dynamic energy at peak airway pressure (coarse pattern) *(Inspiratory volume P = 0.005, Outcome P < 0.001, Interaction = 0.597)*, static energy at peak airway pressure (no pattern) (Inspiratory volume P = 0.002, Outcome P = 0.333, Interaction = 0.618). * P<0.05 between dead and alive pigs in the WITHIN group (see text for description)

Similarly, the 20 pigs ventilated within the limits of inspiratory capacity which finally died had significantly higher dynamic and global energy loads (2.02 ± 1.00 and 2.28 ± 0.86 J) than the 18 pigs that survived (1.20 ± 0.54 J ( *P* = 0.004) and 1.47 ± 0.45 J *( P* < 0.001) (Fig. 5). The static component, in contrast, was not significantly different (0.26 ± 0.46 and 0.27 ± 0.32 J, *P* = 0.10).

**Fig. 6:**
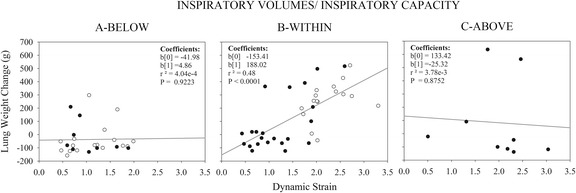
Dynamic strain and increase in lung weight. Relationships between the dynamic strain and the lung weight increase. Pigs were grouped according to normalized inspiratory volume lower (BELOW), within (WITHIN) or higher (ABOVE) than normal inspiratory capacity. They were also divided according to PEEP (0 cmH_2_O, white dots; >0 cmH_2_O, black dots)

As VILI may not be severe enough to be lethal, but sufficient to cause significant lung edema, Fig. 6 illustrates the relationship between the dynamic strain and lung weight changes in the three groups of pigs, independently of survival. In the pigs with end-inspiratory volume above the limits of normal inspiratory capacity (“Above” group), we did not see any significant relationship between dynamic strain and edema. However, this group had a significant relationship between edema formation and time to death, indicating that “time is necessary” for edema to develop (edema = −144.1 + 8.5 × time to death, *R*^2^ = 0.54, *P* = 0.02).

In the pigs with global strain below the lower limit of normal inspiratory capacity (“Below” group), there was no significant increase in lung weight and all, but one survived the whole experiment. In contrast, we found a significant relationship between lung weight increase and dynamic strain applied in the 38 pigs ventilated within the limits of inspiratory capacity (“Within” group). However, lung weight increased without exception only at a dynamic strain higher than 2.

The effects of ventilating these pigs with inspiratory volumes below, within, or above the lung capacity on gas exchange or respiratory mechanics are summarized in Table 2. In the “Below” pigs, although lung weight did not increase, there was a slight but significant deterioration of gas exchange, with a bigger increase in lung elastance. In the “Within” group, the significant increase of lung weight at the end of the experiments was associated with sharp deterioration of gas exchange and marked impairment of lung mechanics. Interestingly, in the “Above” pigs, we found no significant deterioration of gas exchange or lung mechanics at the end of the experiments. Lung elastance, in fact, although abnormally elevated at baseline (because of the extreme ventilator settings) did not get significantly worse during the experiments.

**Table 2:** Gas exchange and respiratory mechanics

	End-inspiratory volumes/inspiratory capacity								
	Below			Within			Above		
	First	Last	*P*	First	Last	*P*	First	Last	*P*
No.	29			38			9		
Lung weight (g)	308 [287–350]	253 [220–275]	0.057	317 [294–349]	483 [301–655]	<0.001	325 [287–340]	242 [220–430]	0.820
PaCO_2_ (mmHg)	38.0 [34.0–43.0]	30.0 [26.0–40.0]	0.005	39.5 [36.0–43.0]	46.5 [39.0–56.0]	0.002	35.8 [34–41.7]	40.8 [35.0–48.8]	0.056
PaO_2_ (mmHg)	234.5 ± 46.0	202.6 ± 58.4	0.020	229.0 ± 40.3	133.3 ± 75.1	<0.001	243.7 ± 27.8	178.8 ± 83.9	0.061
Ers (cmH_2_O/L	32.9 [29.8–38.8]	43.7 [35.6–53.2]	<0.001	33.3 [29.4–45.3]	51.0 [42.8–61.6]	0.005	42.3 [34.9–47.4]	52.9 [41.8–56.8]	0.292
Ecw (cmH_2_O/L)	17.9 [14.5–20.2]	15.9 [14.8–19.8]	0.270	16.4 [13.5–19.0]	15.9 [14.3–19.4]	1.00	14.2 [13.0–19.3]	14.1 [13.7–17.8]	0.808
El (cmH_2_O/L)	15.4 [12.3–22.3]	27.4 [20.2–39.3]	<0.001	19.2 [14.0–30.9]	33.0 [25.9–43.5]	0.018	28.1 [27.6–29.8]	42.7 [35.6–44.6]	0.159
IL-6 (pg/mL)	10.0 [10.0–54.0]	10.0 [10.0–80.0]	1.00	16.5 [10.0–39.0]	185.5 [12.0–449.0]	<0.001	10.4 [5.3–14.3]	14.3 [5.2–694.0]	0.500

Mean ± standard deviation or median (interquartile range) of gas exchange and respiratory mechanics at the beginning (FIRST) and end (LAST) of the experiments. Pigs were grouped according to end-inspiratory volume as lower (BELOW), within (WITHIN), or higher (ABOVE) than normal inspiratory capacity. *P* refers to paired *t* test or Wilcoxon’s Signed Rank Test, as appropriate. Lung weight (available data, 26 below, 38 within, 9 above)

*PaCO*_*2*_ indicates partial pressure of carbon dioxide (29 below, 38 within, 6 above), *PaO*_*2*_ indicates partial pressure of oxygen (29 below, 38 within, 6 above), *Ers* indicates respiratory system elastance (26 below, 37 within, 6 above), *Ecw* indicates chest wall elastance (24 below, 31 within, 5 above), *El* indicates lung elastance (24 below, 31 within, 5 above), *IL-6* indicates serum interleukin 6 (17 below, 28 within, 3 above)

The hemodynamic consequences of ventilating at different end-inspiratory volumes are summarized in Table 3. In the pigs ventilated below inspiratory capacity (“Below” group), the only significant findings at the end of the experiments were small decreases in heart rate and central venous oxygen saturation. In contrast, in the group ventilated with end-inspiratory volumes within inspiratory capacity (“Within” group) at the end of the experiments, pulmonary artery pressure and heart rate were both significantly increased, with significant drops in mean arterial pressure and central venous oxygen saturation. Finally, there were no significant changes in the group ventilated above the upper limits of inspiratory capacity (“Above” group).

**Table 3:** Hemodynamic variables

	End-inspiratory volumes/inspiratory capacity								
	Below			Within			Above		
	First	Last	*P*	First	Last	*P*	First	Last	*P*
No.	29			38			9		
CO (L)	2.1 [1.9–2.8]	2.0 [1.6–2.4]	0.277	2.00 [1.70–2.20]	2.10 [1.50–2.80]	0.569	2.40 [2.35–2.45]	2.05 [1.80–2.75]	0.776
Mean arterial blood pressure (mmHg)	86.4 ± 16.1	77.5 ± 16.3	0.084	78.5 ± 12.3	57. 3 ± 16.4	<0.001	74.7 ± 12.2	77.0 ± 24.7	0.823
Mean pulmonary artery pressure (mmHg)	15.8 ± 5.4	17.8 ± 6.3	0.157	19.8 ± 7.8	25.0 ± 9.8	0.005	23.9 ± 3.4	23.7 ± 4.09	0.911
HR (bpm)	97.4 ± 21.3	83.8 ± 26.1	0.026	108.9 ± 34.6	132.6 ± 49.0	0.021	121.8 ± 31.0	127.2 ± 47.1	0.846
SvO_2_	65.2 ± 8.4	59.8 ± 13.5	0.036	58.0 ± 11.9	48.0 ± 18.7	0.013	57.3 ± 11.2	52.7 ± 8.9	0.372

Mean ± standard deviations or median (interquartile range) of hemodynamic variables at the beginning (FIRST) and end (LAST) of the experiments. Pigs were grouped according to end-inspiratory volume as lower (BELOW), within (WITHIN), or higher (ABOVE) than normal inspiratory capacity. *P* refers to paired *t* test or Wilcoxon’s Signed Rank Test, as appropriate. CO indicates cardiac output (available data, 22 below, 26 within, 4 above); mean arterial blood pressure (29 below, 37 within, 6 above); mean pulmonary artery pressure (26 below, 28 within, 5 above)

*HR* heart rate (27 below, 34 within, 5 above), *SvO*_*2*_ venous oxygen saturation (29 below, 33 within, 6 above)

Table 4 groups the pigs according to their tidal volume (not end-inspiratory volume). In the first group, the median tidal volume (16.3 [12.2–22.6] mL/kg) was below the lower limit of the normal inspiratory capacity (30.9 mL/kg), while in the second group, the median tidal volume (36.6 [34.7–44.7] mL/kg) exceeded the lower limit. Animals in the first group that survived had significantly lower PEEP than the animals that died.

**Table 4:** Respiratory mechanics in pigs grouped according to tidal volume

	Tidal volume below the lower limit of normal inspiratory capacity			Tidal volume above the lower limit of normal inspiratory capacity		
	Alive	Dead	*P*	Alive	Dead	*P*
No.	40	7		9	20	
Tidal volume (mL/kg)	16.0 [13.0–23.5]	16.7 [10.0–21.1]	0.591	34.7 [32.5–38.6]	37.0 [35.6–46.3]	0.045
PEEP (cmH_2_O)	5 [0–10]	20 [6–26]	0.010	4 [0–5]	0 [0–6]	0.698
PEEP/ZEEP	24/16	6/1	0.165	5/4	8/12	0.436
Inspiratory volume (mL/kg)	23.8 [16.5–43.3]	48.9 [37.8–55.3]	0.009	41.1 [34.7–47.8]	46.3 [36.8–62.8]	0.334
Global strain	1.74 [0.90–2.21]	2.82 [2.44–4.18]	<0.001	2.34 [2.02–2.44]	2.64 [2.21–3.17]	0.085
Dynamic strain	0.87 [0.65–1.34]	0.97 [0.73–1.40]	0.630	1.93 [1.84–2.02]	2.34 [1.98–2.58]	0.005
Static strain	0.00 [0.00–1.15]	2.18 [1.11–2.97]	0.005	0.48 [0.00–0.63]	0.00 [0.00–0.72]	0.698
FRC (mL)	397 ± 102	361 ± 73	0.370	387 ± 78	382 ± 91	0.880

Mean ± standard deviations or median (interquartile range) or occurrences of respiratory mechanics variables in pigs grouped according to their tidal volume (not end-inspiratory volume) in relation to normal inspiratory capacity (below or above the lower limit). Pigs were then grouped according to outcome. *P* refers to paired *t* test or Wilcoxon’s Signed Rank Test, as appropriate, for continuous variables and the chi-square test for categorical variables

*PEEP* indicates positive end-expiratory pressure, *ZEEP* indicates zero end-expiratory pressure, *FRC* indicates functional residual capacity

## Discussion

In this study, we considered VILI in the framework of lung anatomical constraints and physical forces. To do so, we had to rely on variables such as strain, stress, and mechanical energy instead of tidal volume/kilogram ideal body weight and airway pressures (plateau and PEEP). Strain measures the actual distortion of the lung: for the same tidal volume/kilogram, the strain may be completely different depending on the size of the “baby lung” [18, 19]. Likewise, the same airway pressure may result in widely differing transpulmonary pressures (stress), depending on the relationship between lung and chest wall elastances [20]. The initial trigger of stress and strain, however, is the force applied to the extracellular matrix times its displacement, which equals the product of pressure times delta-volume (*P* × dV). The cyclic energy loads applied at a given frequency (power) trigger the VILI, which may be seen in this context as a sort of “fatigue” of the extracellular matrix, similar to material “fatigue” [21–24]. We believe that the energy/power concept, which encompasses stress, strain, frequency, and flow rate, explains the different effects of tidal volume (dynamic), PEEP (static), and the physical thresholds conditioning the appearance of VILI.

### Inspiratory capacity

Referring to inspiratory capacity, we used a range derived from the whole population instead of a single value to account for physiological variability (inspiratory capacities were normally distributed in our population). The resulting distribution of inspiratory capacity is of the same order of magnitude as in humans (≈1/3 of the mean) [25]. The ratio of inspiratory capacity (TLC-FRC) to resting lung volume (FRC) in these pigs was 2.6 ± 0.6, similar to mice (≈2–2.3, TLC≈1–1.5 mL FRC≈0.3–0.5 mL) [26–28], rats (≈2–3, TLC≈10 mL, FRC≈2.5–3 mL) [28, 29], and normal humans (≈2.2, TLC≈7000 mL, FRC≈2200 mL) [28, 30]. Therefore, in the species most commonly used to study VILI, the limits of physical expansion of the lung are 2–3 times the FRC, i.e., strain between 2 and 3.

While the strain to reach inspiratory capacity is similar within the species, the associated stress (equal to the transpulmonary pressure) differs widely, due to differences in specific elastance. As an example, the specific elastance (transpulmonary pressure necessary to double the FRC) is approximately 4 cmH_2_O in rats [31], 6 cmH_2_O in pigs [14], and 12 cmH_2_O in humans [20]. Therefore, the stress associated with inspiratory capacity may vary widely within a species, and this must be kept in mind when experimental research is translated to the human being.

### Volume and pressure thresholds for ventilator-induced lung injury

Our findings indicate that the volumetric threshold for VILI coincides with the anatomical limits of lung expansion. In the pigs ventilated with end-inspiratory volumes below the lower limits of inspiratory capacity, we could not find any real increase of lung weight, suggesting there was no VILI. The slight deterioration of gas exchange and the increased lung elastance in this group were very likely due to dependent atelectasis after 54 h of anesthesia and paralysis [32–34]. The “stress at rupture” appeared in the “Above” group where the end-inspiratory volume exceeded the upper limit of inspiratory capacity, probably overcoming the collagen’s ultimate strain [35]. In the “Within” group, at lower stress and strain, the appearance of edema was proportional to tidal volume (Fig. 6). Strain at the lower limit of inspiratory capacity in our experiments was 1.4, and the associated stress averaged 10.2 cmH_2_O, corresponding to an airway pressure of 20.3 cmH_2_O. Below these values, no VILI occurred (“Below” group).

While the pressometric values cannot be translated as such to the human being due to differences in specific elastance, the strain threshold we found (≈2.6) was similar to the harmful lethal strain found in mice, rats, rabbits, pigs, and sheep [29].

### Dynamic versus static strain

How the volumetric/pressometric thresholds are reached seems important in relation to the appearance and severity of VILI. Webb and Tierney [8], in their seminal experiments on rats ventilated at 45 cmH_2_O airway pressure for nearly 1 h, found huge damage when global strain was 100 % dynamic (tidal volume) but only minimal damage when the same global strain was 66 % dynamic (tidal volume) and 33 % static (PEEP). The same observations were reported by Dreyfuss and coworkers [3]. We recently found that global strain of 2.5 in pigs was lethal if 100 % dynamic (tidal volume) but safe if 25 % dynamic and 75 % static [15]. Similarly, the dynamic strain induced damage in cell cultures while the same strain in static conditions did not [36]. The bulk of data so far, therefore, leads to the common belief that dynamic strain (tidal volume) is harmful while static strain (PEEP) is “protective”.

It is convenient, in our opinion, to interpret these results taking into account the energy load associated with dynamic and static strain. The energy load is the integral of *P* × dV along the PV curve. If an “excess” of energy is loaded onto the system, we can expect the unrecovered energy to be sufficient to break the molecular bonds of the polymers of the extracellular matrix [37–39], to detach endothelial [21] and epithelial cells [40] from the basement membrane, and to fracture the capillary walls [41]. Alteration of the extracellular matrix with the appearance of polymers with lower molecular weight, combined with capillary micro-fractures, may activate the inflammatory reaction [42] and micro-hemorrhage, leading to the extracellular edema typical of VILI.

The behavior of PEEP is more complex. PEEP provides increased continuous tension to the extracellular matrix which accumulates energy equal to (PEEP × PEEP volume)/2. Further energy is added when tidal volume (dynamic cyclic energy) is superimposed on the PEEP to reach a given end-inspiratory volume. Therefore, if the end-inspiratory volume is the same, with or without PEEP, the energy is lower in the presence of PEEP than without it (Fig.3), as in our group of “Within” animals. In contrast, if the same tidal volume is provided with or without PEEP, the energy with PEEP will be higher and potentially harmful, as presumably occurred in our “Above” group (Table 4). Therefore, considering the energy associated with a given ventilatory mode, it is clear that the independent variable for VILI is dynamic strain (tidal volume), while PEEP is “protective” as far as it is associated with a lower tidal volume, as in the “Within” group. Otherwise, it has no effect, as in the “Below” group, or is even harmful, as in the “Above” group.

Finally, when considering energy, we cannot ignore the respiratory rate. All our experiments were done at 15 bpm, but it is always possible that at a lower rate, there would be less severe damage—or none at all—for the same exposure time.

### Translation to a clinical scenario

Taken with the appropriate caution, our findings may be of some interest when approaching mechanical ventilation in human ARDS. This syndrome involves reduced FRC—the baby lung [18, 19]. As the specific elastance of the baby lung is usually normal [20], we may expect the volumetric threshold, if similar to pigs, to be ≈2.6 ± 0.6 times higher. As an example, in a 70-kg man with severe ARDS, if the baby lung is around 300 mL, ignoring for simplicity’s sake 10–15 % of recruitability, the inspiratory capacity would be 780 mL. At 12 mL/kg body weight, the tidal volume would be 840 mL, within the range of inspiratory capacity. If we refer to the lower limits of inspiratory capacity ((2.6–2×0.6×300 = 420 mL), even the 6 mL/kg ventilation would be close to the volumetric threshold. To translate the pressometric threshold of pigs to humans, the pressure observed in pigs at inspiratory capacity should be approximately multiplied by 2, to take account of the difference in specific elastance.

In addition, we must remember the “inhomogeneity” factor. We estimated that the uneven distribution of volumes and pressure could induce local stress/strain about double that computed for the whole lung [43]. Therefore, in an ARDS patient, knowing the volumes and transpulmonary pressure, and taking account of the inhomogeneity factor, we can forecast whether in a given patient low tidal volume ventilation will still be safe or alternative forms of respiratory assistance, such as an artificial lung, should be employed.

The driving pressure has been recently advocated as the variable most related to VILI [44]. The driving pressure, however, should be considered in relation with the chest wall elastance, the lung size, homogeneity, and gas flow rate. Depending on the combination of these variables, the same driving pressure may be “lethal or innocent”. In summary, our study confirms that a single variable cannot explain the complexity of VILI. The concept of energy and power, encompassing several different variables, may reconciliate some of the contradictions present in the literature.

Similar concepts may be applied when considering the harm of high tidal volume ventilation during anesthesia or ventilating ICU patients with uninjured lungs. The primary difference between a normal lung and the ARDS lung is that, for a given tidal volume, the stress and strain are greater in ARDS [20] due to the inhomogeneities of the lung parenchyma [43] and the size of the “baby lung”. However, also the “normal” lung in anesthesia or the uninjured lung during mechanical ventilation [45] may present inhomogeneity with mal-distributed stress due to comorbidities or aging. Current data from large randomized trials and meta-analyses on the effects of mechanical ventilation on pulmonary complications during anesthesia show that the number of complications increases with tidal volume and driving pressure, with increasing tidal volume at the same PEEP [46–48], increasing tidal volume at low PEEP [46, 48-50] and increasing PEEP at constant tidal volume [51]. All these results are in line with the model presented in this paper.

### Limitations

We recognize that the largest threat to the interpretation of our data hinge on the reliance of data collected in previous studies. Therefore, biases arising from the use of historical data may be present. However, the experimental animals were all of the same breed, had similar weight, were coming from the same farm, and experiments were conducted by the same people in the same experimental setting. In addition, we believe that a prior data publication does not compromise the novelty of a study if it goes beyond a descriptive analysis of the data and report new scientific findings [52].

## Conclusions

We found that the threshold of VILI in the healthy lung is the region defining the inspiratory lung capacity, i.e., the anatomical limits of lung expansion. When the inflated volume is below the threshold, VILI does not occur, and if it is within the limits, it appears as a main function of its dynamic component. If it exceeds the total lung capacity, stress at rupture occurs. A unifying explanation is that the trigger for VILI is an excessive energy/power load, which encompasses pressures, volume and—though not tested in the present study—respiratory rate and flow. PEEP, not associated with energy input, appears to prevent VILI if the tidal volume is lower. Otherwise, PEEP may be harmful as it just boosts inflation closer to the total lung capacity.

## Abbreviations

ANOVA: analysis of variance
ARDS: acute respiratory distress syndrome
CT: computed tomography
FRC: functional residual capacity
HU: Hounsfield units
PEEP: positive end-expiratory pressure
PV: pressure-volume
*P* × dV: pressure times volume change
SD: standard deviation
TLC: total lung capacity
TV: tidal volume
UIP: upper inflection point
VILI: ventilator-induced lung injury
